# Biological single-particle imaging using XFELs – towards the next resolution revolution

**DOI:** 10.1107/S2052252518015129

**Published:** 2018-10-31

**Authors:** Dominik Oberthür

**Affiliations:** a Center for Free-Electron Laser Science, Deutsches Elektronen-Synchrotron DESY, Notkestrasse 85, 22607 Hamburg, Germany

**Keywords:** Rayleigh scattering, XFELs, aerosol injection, single-particle imaging

## Abstract

Better injectors resulting from careful iterative optimization used at high repetition XFELs in combination with better detectors and further developed algorithms might, in the not so distant future, result in a ‘resolution revolution’ in SPI, enabling the molecular and atomic imaging of the dynamics of biological macromolecules without the need to freeze or crystallize the sample.

Since the publication of computational simulations (Neutze *et al.*, 2000 ▸) that predicted the possibility of deriving atomic resolution structural information of biological macromolecules from single-particle imaging (SPI) (Barty, 2016 ▸) at room temperature when using ultra-short (few fs long) X-ray pulses, SPI has been one of the main scientific drivers and one of the most used cases to obtain funding for the construction of XFEL sources. This rapidly led to the construction and successful operation of XFELs that in principle could be used for SPI. Especially for the European XFEL and for the LCLS, it is clear that SPI of biological macromolecules is one of the main topics of research with dedicated beamlines that, at least initially – before the success of serial femtosecond crystallography (SFX) (Chapman *et al.*, 2011 ▸) – were designed for this purpose (CXI at LCLS and SPB at XFEL).

## 1. SPI – growing in the shadow of SFX and cryoEM   

While SFX resulted in new structures (Colletier *et al.*, 2016 ▸; Kang *et al.*, 2015 ▸; Redecke *et al.*, 2013 ▸) that could not be obtained from crystallography at room temperature, and both revived and revolutionized time-resolved crystallography (Nogly *et al.*, 2018 ▸; Olmos *et al.*, 2018 ▸; Pande *et al.*, 2016 ▸), the initial results from biological SPI (Seibert *et al.*, 2011 ▸; Ekeberg *et al.*, 2016 ▸) showed that the X-ray intensity from XFELs, the available detectors, and techniques to introduce the sample into the focused X-ray sampling position, were all insufficient to obtain (near) atomic resolution structural information from biological macromolecules. Moreover, at the same time as the first SPI experiments were carried out, the ‘resolution revolution’ in cryo transmission electron microscopy (cryoEM) (Subramaniam *et al.*, 2016 ▸) became evident, achieving the goal of near atomic resolution single-particle imaging. While experimental beam time at XFELs has always been difficult to obtain, especially for methods development (understandable considering both the construction and running costs of such large-scale facilities), developments in cryoEM benefited from the relatively large number of high-end instruments. A lot of researchers could improve many things in parallel, with much less immediate pressure to obtain high-impact research results. New developments could be implemented rapidly by multiple groups, and thus progress in the past five or so years has been much faster than previously, and more spectacular. However, it is frequently overlooked just how long it took from the beginning of the development of single-particle cryoEM (Nogales, 2016 ▸) until new detectors, better instruments and better software finally made the ‘resolution revolution’ possible.

SPI at XFELs is still a very young field of science where, just as in cryoEM some 30 years ago, experimental obstacles, rather than theoretical boundaries, are the limiting factors. Indeed, some of the experimental problems are similar to the challenges cryoEM had to face for many years. For instance, the development of X-ray detectors equivalent in performance to that of the direct electron detector used in cryo-EM has yet to be fully realized. Other problems are rather specific to the nature of the generic SPI experiment: the number of X-ray pulses delivered by the FEL per second, the intensity of each pulse, shot-to-shot variations in pulse intensity and X-ray energy, sample delivery, the nature of the sample and associated radiation damage effects, and even the background noise/scatter introduced from various sources, all remain challenges in FEL experiments.

To systematically approach these challenges the ‘SPI roadmap at LCLS’ (Aquila *et al.*, 2015 ▸) was proposed and built up as an international collaboration known as the ‘SPI initiative’. The ‘SPI initiative’ has received several slots of experimental beam time at both the CXI and the AMO experimental stations at LCLS, and this large international collaboration has been able to work systematically on the experimental limitations of SPI. The collected data have been published (Rose *et al.*, 2018 ▸; Reddy *et al.*, 2017 ▸; Kurta *et al.*, 2017 ▸; Munke *et al.*, 2016 ▸) and released to the CXIDB (Maia, 2012 ▸), and thus have been available for use by other researchers to develop algorithms for SPI further. Most importantly, this systematic approach can reveal the real bottlenecks in SPI (apart from more FEL pulses per second, which will improve in any case with SPI at the European XFEL and at LCLS-II). It turns out that one of these is – considering the need to minimize the background noise – the ratio of ‘X-ray pulses hitting a molecule of interest’ to ‘the X-ray pulses available’ (the so-called ‘hit rate’). A low hit rate has two main negative results: a sample that may be difficult and expensive to produce is essentially wasted and, more importantly, it limits the useful amount of data that can be collected in a certain time. And to retrieve high-resolution information from small biological objects, such as single molecules of a protein, a lot of data need to be collected (Sun *et al.*, 2018 ▸).

To improve SPI to the point where it is able to reach the goal of molecular and atomic imaging of the dynamics of biological macromolecules at room temperature, one thus needs to understand and improve the way the sample is introduced into the X-ray focus. For SPI basically three different concepts have been presented to bring the sample into the X-ray focus: (1) aerosol injection (Hantke *et al.*, 2018 ▸), (2) liquid jets and (3) fixed targets (Seuring *et al.*, 2018 ▸), of which aerosol injection is by now the most used. The advantages of aerosol injection are the rapid replenishment of sample, that the sample is surrounded by a layer of water, which shields it from the hostile environment in the experimental chamber, and that this layer is thin enough, to not contribute too much to background scattering. The disadvantage is the low hit rate, especially for a small (≪1 µm) X-ray focus, which in turn is needed if one aims for the highest possible intensity. The hit rate at a given X-ray focus is determined by the particle density that the injector can deliver.

## 2. Better sample delivery for better structures   

**Figure 1:**
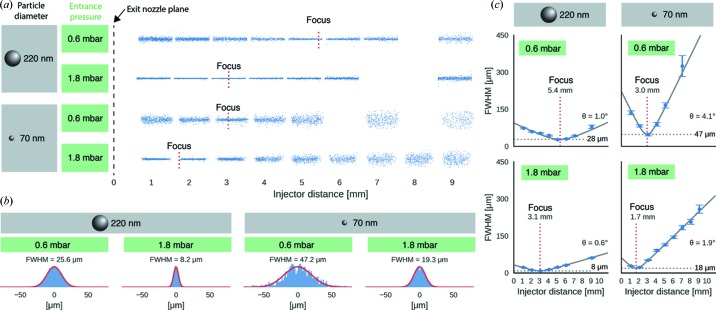
Particle-beam focusing as function of entrance pressure and particle diameter [reproduced from Hantke *et al.* (2018 ▸)]. (*a*) Blue dots represent measured particle positions of injected polystyrene spheres (70 nm and 220 nm in diameter) at entrance pressures of 0.6 mbar and 1.8 mbar, respectively. Gaps are a result of combining the data from measurements at fixed injector distances without overlap of the fields of view. The positions of the focus planes are indicated by dotted red lines. (*b*) Measured particle-beam profiles (blue histograms) in the particle-focus plane were approximated by Gaussian functions (red lines). (*c*) The evolution of the particle-beam width (blue circles) was approximated with a Gaussian-beam model (black solid lines). The model is parameterized by a divergence angle θ, the beam waist (gray dashed lines) and the position of the focus plane (red dotted lines).

Hantke *et al.* (2018 ▸) now present an elegant way to characterize aerosol injectors and to visualize the particle density in the aerosol at a given point – even for particles as small as 40 nm in diameter. In their work published in this issue of **IUCrJ**, they used a Rayleigh-scattering microscopy set-up to understand better how aerosol injectors work. In particular they looked at how particle density at a certain point depends on particle size and aerodynamic lens entrance pressure (Fig. 1 ▸). Moreover, the speed of particles coming out of the injector was analyzed and found to be sufficient to cope with the full repetition rate of the European XFEL, where subsequent pulses are only separated by 200 ns. Their set-up is also capable – if properly calibrated – of estimating the size of the detected particles in the aerosol. This is very important for improving the sample and for optimizing sample delivery for XFEL experiments (Hantke *et al.*, 2018 ▸).

With this technique, one can now rationally improve the design of the most used sample delivery method for SPI at XFELs. It is possible to test new injector designs, to test new samples before an experiment, and to iteratively optimize both of these for best possible injection results. The Rayleigh-scattering microscopy set-up is thus an important step towards removing the sample delivery bottleneck in single-particle imaging of biological specimens at XFELs. Better injectors resulting from careful iterative optimization used at high repetition XFELs in combination with better detectors and further developed algorithms might, in the not so distant future, result in a ‘resolution revolution’ in SPI, enabling the molecular and atomic imaging of the dynamics of biological macromolecules without the need to freeze or crystallize the sample.
